# Replacing Ambulatory Surgical Follow-Up Visits With Mobile App Home Monitoring: Modeling Cost-Effective Scenarios

**DOI:** 10.2196/jmir.3528

**Published:** 2014-09-22

**Authors:** Kathleen A Armstrong, John L Semple, Peter C Coyte

**Affiliations:** ^1^Division of Plastic and Reconstructive SurgeryDepartment of SurgeryUniversity of TorontoToronto, ONCanada; ^2^Institute of Health Policy, Management and EvaluationUniversity of TorontoToronto, ONCanada; ^3^Department of Ambulatory SurgeryWomen's College HospitalToronto, ONCanada

**Keywords:** cost-effectiveness, ambulatory surgical procedures, mobile apps, ambulatory monitoring

## Abstract

**Background:**

Women’s College Hospital (WCH) offers specialized surgical procedures, including ambulatory breast reconstruction in post-mastectomy breast cancer patients. Most patients receiving ambulatory surgery have low rates of postoperative events necessitating clinic visits. Increasingly, mobile monitoring and follow-up care is used to overcome the distance patients must travel to receive specialized care at a reduced cost to society. WCH has completed a feasibility study using a mobile app (QoC Health Inc, Toronto) that suggests high patient satisfaction and adequate detection of postoperative complications.

**Objective:**

The proposed cost-effectiveness study models the replacement of conventional, in-person postoperative follow-up care with mobile app follow-up care following ambulatory breast reconstruction in post-mastectomy breast cancer patients.

**Methods:**

This is a societal perspective cost-effectiveness analysis, wherein all costs are assessed irrespective of the payer. The patient/caregiver, health care system, and externally borne costs are calculated within the first postoperative month based on cost information provided by WCH and QoC Health Inc. The effectiveness of telemedicine and conventional follow-up care is measured as successful surgical outcomes at 30-days postoperative, and is modeled based on previous clinical trials containing similar patient populations and surgical risks.

**Results:**

This costing assumes that 1000 patients are enrolled in bring-your-own-device (BYOD) mobile app follow-up per year and that 1.64 in-person follow-ups are attended in the conventional arm within the first month postoperatively. The total cost difference between mobile app and in-person follow-up care is $245 CAD ($223 USD based on the current exchange rate), with in-person follow-up being more expensive ($381 CAD) than mobile app follow-up care ($136 CAD). This takes into account the total of health care system, patient, and external borne costs. If we examine health care system costs alone, in-person follow-up is $38 CAD ($35 USD) more expensive than mobile app follow-up care over the first postoperative month. The baseline difference in effect is modeled to be zero based on clinical trials examining the effectiveness of telephone follow-up care in similar patient populations. An incremental cost-effectiveness ratio (ICER) is not reportable in this scenario. An incremental net benefit (INB) is reportable, and reflects merely the cost difference between the two interventions for any willingness-to-pay value (INB=$245 CAD). The cost-effectiveness of mobile app follow-up even holds in scenarios where all mobile patients attend one in-person follow-up.

**Conclusions:**

Mobile app follow-up care is suitably targeted to low-risk postoperative ambulatory patients. It can be cost-effective from a societal and health care system perspective.

## Introduction

Technology is identified as an opportunity to constrain the growth in health care costs and eliminate barriers due to distance [1]. In Ontario (Canada), specialized surgical services tend to be concentrated within metropolitan areas. This results in many patients having to travel great distances to receive care. Women’s College Hospital (WCH) in Toronto offers specialized ambulatory surgical procedures, including breast reconstruction following mastectomy for breast cancer. Ambulatory surgery means that the patient goes home within 24 hours of surgery and comes back at a later date for follow-up care. The average ambulatory breast reconstruction patient travels 76 km from home to hospital, with the furthest patient coming from 540 km away. Similarly in Ontario, 23% of all orthopedic surgery patients leave their local health care catchment to receive care [2].

Patients not only travel to receive care, they also travel to receive follow-up care. In an ambulatory (or outpatient) surgery patient population, travel for postoperative follow-up seems superfluous as the chance of postoperative complication is exceedingly low. This is because of advancements in surgery and rigorous patient selection. In general, ambulatory surgery is largely reserved from the treatment of American Society for Anesthesia (ASA) class I and II patients [3]. These patients are considered healthy or with mild systemic disease, respectively. Complication rates in this subset of breast reconstruction patients are approximately 5% [4]. If a complication occurs, it is typically a minor skin infection or wound dehiscence. Rarely (<1%), a hematoma requiring surgical evacuation may occur. These types of complications occur suddenly and present to the emergency department.

**Figure 1:**
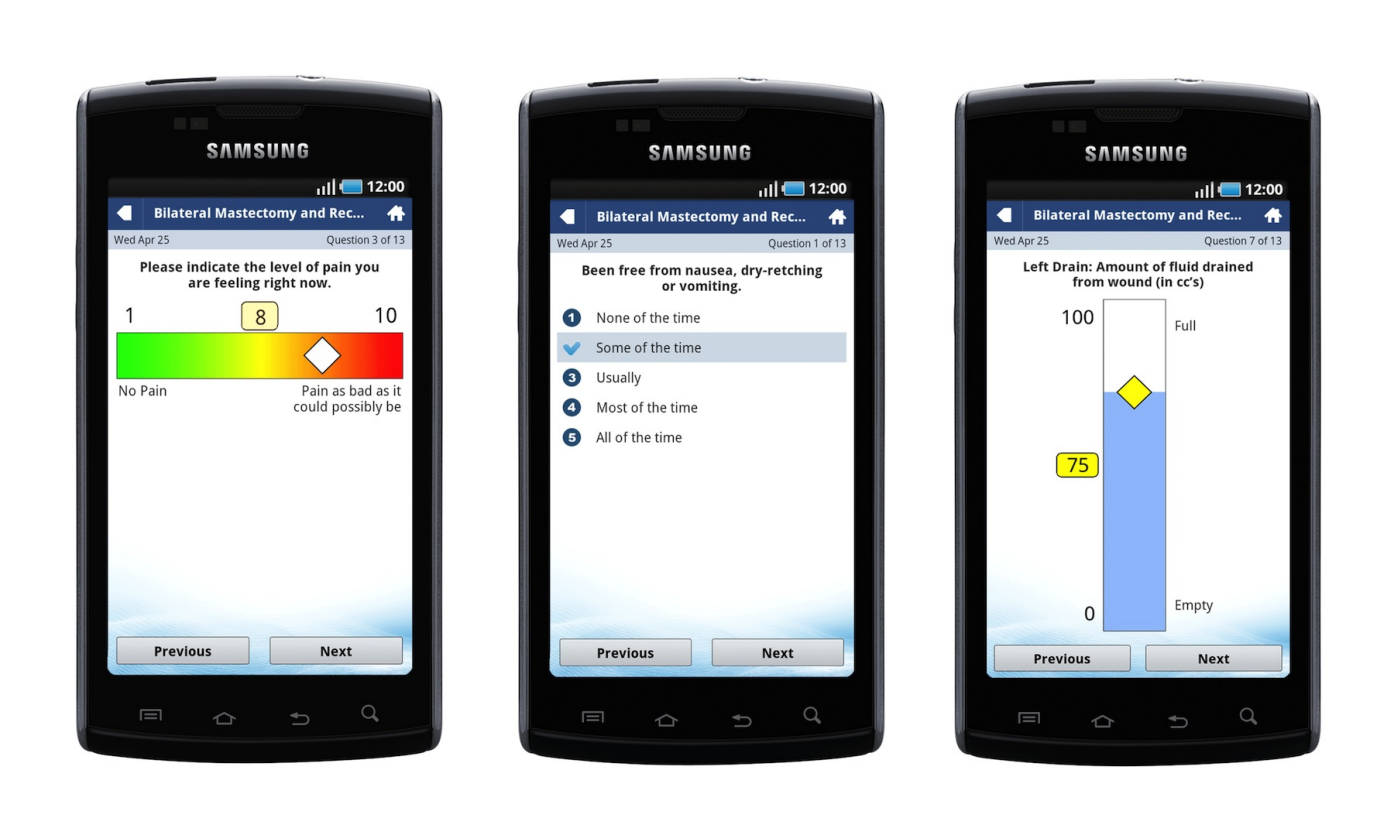
The mobile app user interface.

Finding solutions to limit unnecessary burden of care associated with travel is a worthwhile goal in any patient population. However, it is particularly important in patient populations where rurality and lower socioeconomic status are known barriers to breast reconstruction [5]. For these reasons, WCH has completed a feasibility study using a mobile app (QoC Health Inc, Toronto) to support postoperative care in breast reconstruction patients. This mobile app raises the bar by combining validated quality of recovery questionnaires and surgical site photos submitted at the patients’ convenience in an asynchronous manner (see Figure 1). The study suggests that mobile app follow-up care adequately detects postoperative complications and eliminates the need for in-person follow-up care. This is concordant with other postoperative telemedicine studies [3,6,7].

Previous studies have found that after a tonsillectomy or adenoidectomy, telephone follow-up care with standardized questionnaires is as safe as standard follow-up care and offers considerable cost reduction and patient convenience [3]. Similar telephone follow-up has also been used successfully in elective open hernia repairs, laparoscopic cholecystectomy, and curative breast cancer surgery [6,7]. Others have shown that planned outpatient appointments after uncomplicated surgery are neither necessary nor cost-effective [8]. A “no planned follow-up” saves money for hospitals and patients [8].

Patients are highly satisfied with telephone follow-up. Still, there are some glaring disadvantages when telephone follow-up care is compared to mobile app follow-up care. Telephone follow-up relies on synchronous communications between patients and health care providers. Studies report between 15 and 27% of patients were unreachable by phone after multiple attempts to reach postoperative patients [6,9]. It is also heavily dependent on labor costs as a nurse or other health care worker is designated to call, collect, and relay the questionnaire information to the primary surgeon. This makes the questionnaire data more expensive to relay when compared to a mobile app, which transmits directly from patient to surgeon. Similarly, “no planned follow-up” is poorly received by patients and providers who value continuity of care [8]. In this way, mobile app follow-up care offers an optimal middle ground between conventional in-person follow-up care, telephone follow-up care, and no planned follow-up care.

The proposed project provides breast reconstruction patients with timely contact with their surgeon from the comfort of their home. This technology has the potential to address wait times by freeing up specialty surgeon clinic time so that they may engage in new consultations and attend to patients in the emergency department. The first step in more widespread implementation involves demonstrating cost-effectiveness.

## Methods

This study used method recommendations from international health technology assessment (HTA) agencies for economic evaluations to develop a model for comparing mobile app follow-up care with in-person follow-up visits [10]. Inputs and outputs were chosen based on relevance to the decision-making perspective of the economic evaluation [10]. Cost data were derived from WCH breast reconstruction patient administrative data and QoC Health Inc mobile app billed costs. This is in keeping with HTA agency recommendations [10]. A societal perspective was adopted wherein all costs were assessed irrespective of the payer [11]. Again, this perspective was chosen based on HTA agency recommendations. This recommendation is meant to improve comparability and consistency across studies [12]. The patient/caregiver, health care system, and externally borne costs are calculated within the first postoperative month. The results are also presented using a narrower health care system perspective that may be of key interest to health administrators and policy decision makers. The effectiveness of mobile app and conventional follow-up care was measured as successful surgical outcomes at 30-days postoperative. Successful surgical outcome was clearly defined as a “surgical patient not requiring medical or surgical intervention related to the original surgery within the first 30-days postoperative”. This was deemed an important outcome where no meaningful difference in health-related quality of life (HRQL) between mobile app and in-person follow-up care has been demonstrated [13]. The 30-day time horizon was chosen based on literature surrounding postoperative complications in the first 30-days [14]. It was felt to be long enough to capture all relevant costs and benefits of mobile app and in-person follow-up care. Effectiveness data were derived from clinical studies from similar ambulatory patient populations. Model parameters were input into TreeAge software.

Cost data were collected from a societal perspective using a micro-costing approach advocated for by HTA agencies [10]. All cost data were based on 2013/2014 estimates.

In-person follow-up costs incurred by the health care system include employees, compensation, drugs, surgical instruments and supplies, equipment, and other (eg, linens, telephone charges, general supplies), specialized breast center clinical assistant compensation, resident compensation, and physician fee (see Table 1). WCH provided per patient clinic costs and Ontario Health Insurance Plan (OHIP) billing codes were used to determine physician fees. Physician payment methods were verified through the hospital to ensure double counting did not occur. In keeping with cost-effectiveness analysis, non-recoupable or sunk costs were not included in the in-person follow-up arm [15].

Mobile app follow-up costs incurred by the health care system include the start-up fixed costs such as: health center setup, design/setup of procedure protocols, and training of hospital staff. The start-up costs were divided over the number of patients served over the useful lifespan of smartphone technology, which was conservatively estimated at 5 years. Current e-assessment OHIP physician billing codes are limited. There is currently no OHIP billing code for surgical e-assessments. In the future, we assume that billing codes will exist and so we applied a fee based on actual OHIP telemedicine follow-up fees. The variable costs for mobile app follow-up care included software, licensing, and technical support. The bring-your-own-device (BYOD) model variable cost was $3.50 CAD per patient per day. This costing assumes that 1000 patients are enrolled in BYOD mobile app follow-up per year based on a QoC Health Inc business model that enrolls hospitals.

In-person follow-up costs incurred by the patient included foregone patient leisure time, the wage of a caregiver, and travel and parking costs associated with follow-up visits. We determined foregone leisure costs based on labor force participation rates and age-sex adjusted average Ontario wages. Labor force non-participants were assigned an Ontario homemaker’s wage [16]. We presumed that a caregiver equivalent would be present at the first follow-up visit, and assigned a homemaker wage ($11.28 per hour) to that person [16]. This is considered a conservative estimate of true caregiver costs because most patients bring their partner with them to clinic, and those individuals would earn higher average age/sex adjusted wages. The hourly rates were multiplied by the travel time and length of the clinic visit. The clinic time was assumed to be 1 hour to include time to park, register, and meet with the health care team. Travel time and costs estimates were based on actual breast reconstruction patient distance data from home postal code to WCH. Canadian Automobile Association (CAA) Ontario-based average costs per km driven were used to calculate transportation costs. The number of clinic visits was averaged at 1.64 visits over the first postoperative month based on actual attendance by breast reconstruction patients at WCH.

Mobile app follow-up costs incurred by the patient were modeled based on a BYOD format, in which the patient loads the app on to their own mobile phone. Costs included the foregone leisure time to submit follow-up data and the cost of data submission. Each submission takes approximately 3 minutes to enter and submit. In the feasibility study, patients were asked to submit monitoring information once daily for the first 2 weeks and then once weekly for the next 2 weeks. Leisure time was not interrupted by the submission of a mobile app follow-up; therefore, there was minimal sacrifice. Each submission (including survey information and photo) used approximately 0.35 MB of data. In Ontario, 2 GB of data can be purchased for $45 CAD [17]; therefore, data costs were negligible. Patient training sessions were held while patients waited for their preoperative appointment. There were no additional patient costs associated with this time.

This modeling study used telephone follow-up studies to determine the effectiveness of mobile app follow-up when compared to in-person follow-up care. Telephone and mobile app follow-up are considered to transmit the same questionnaire-based data from patient to provider. HTA agencies recommend conducting a systematic review of the literature on key model inputs including effect data; however, clinical trials and observational studies can be used to obtain effect data if they more appropriately represent the model of interest [10]. A recent article in BMC Health Services Research systematically reviewed telephone consultations in place of face-to-face outpatient consultation for patients discharged from hospital following surgery. It reported low methodological quality and dissimilar outcomes [18]. None of the articles included in the review captured patient populations or outcomes that were comparable to the patient populations and outcomes modeled in this study. The lack of comparative data reflects the fact that type of follow-up care (mobile app, telephone, or in-person) does not impact the chance complication. For this reason, baseline equivalence in effect was modeled between the two groups. This assumption is supported by large observational studies following laparoscopic cholecystectomy, inguinal and paraumbilical hernia repair, other hernia repair, varicose vein surgery, circumcision, excision of subcutaneous lesions, carpal tunnel release, and appendectomies [6,9]. These studies found that structured postoperative telephone questionnaires conducted between 2 and 6 weeks were a safe alternative to in-person follow-up care [6,9]. Telephone questionnaire-based follow-up adequately detected patients that required further in-person assessment (5-11% of all patients) [6,9]. These studies contain similar patient populations, procedural variation, and surgical risks when compared to ambulatory breast reconstruction patients.

Three types of sensitivity analyses were performed to determine their effects on costs and outcomes. A scenario analysis was conducted for variations in the number of in-person clinic visits and crossover from mobile app follow-up to in-person follow-up. A two-way sensitivity analysis varied patient wage and mobile app follow-up effect. Additionally, a probabilistic sensitivity analysis was performed to account for uncertainty in the distribution of patient, caregiver, and clinic costs as well as uncertainty in effects (ie, complication rates).

## Results

### Overview

The results of this analysis are summarized in Table 1. The total cost difference between mobile app and in-person follow-up care was $245 CAD ($223 USD based on the current exchange rate), with in-person follow-up being more expensive ($381 CAD) than mobile app follow-up care ($136 CAD). This takes into account the total of health care system, patient, and external borne costs. If we examine health care system costs alone, in-person follow-up was $38 more expensive than mobile app follow-up care (please see Table 1). The baseline difference in effect was modeled to be zero based on the WCH feasibility study, as well as other ambulatory telephone follow-up studies. An incremental cost-effectiveness ratio (ICER) is not reportable in this scenario. An incremental net benefit (INB) is reportable, and reflects merely the cost difference between the two interventions for any willingness-to-pay value (INB=$245 CAD).

**Table 1:** Cost breakdown.

In-person follow-up			Cost (CAD $)	Mobile app follow-up	Cost (CAD $)
**Health care system costs**					
	**Fixed costs**				
		Compensation	103.74	Health center setup	1.39
		Equipment	2.16	Design/setup procedure protocol	6.94
				Training	0.44
	**Variable costs**				
		Drugs	0.21	Platform licensing, accounts	42.00
		Other (Linens)	3.83	Standard support	43.05
		Clinical assistant (10 min)	10.25	Infrastructure hosting	19.95
		Surgeon fee	43.46	Surgeon fee	22.00
		Resident	10.56		
		Health care system costs subtotal (per patient per 1.64 visits over 30 days)	$174	Health care system costs subtotal (per patient per 30 days monitoring)	$136
**Patient costs**					
	**Variable costs**				
		Patient leisure time	102.24	Patient leisure time	negligible
		Caregiver wage	33.84	Data (approx. 350 kB per transmission with photo)	negligible
		Travel (to and from clinic)	38.11		
		Parking	32.80		
		Patient costs subtotal (per patient per 1.64 visits over 30 days)	$207	Patient costs subtotal (per patient per 30-day monitoring)	negligible
**Total societal costs^a^**					
		Per patient per 1.64 visits over 30 days	$381	Per patient per 30-day monitoring period	$136

^a^Total societal costs = health care system costs subtotal + patient costs subtotal

### Scenario Analysis: Societal Perspective Costs With Varying Number of In-Person Visits in the First Month Postoperative

The number of in-person follow-up visits was set to a minimum value and compared to the costs of mobile app follow-up. This sensitivity analysis demonstrates that even at only 1 in-person visit per patient over the first month postoperative, mobile app follow-up care is less costly from a societal perspective. From a societal perspective, mobile app follow-up care remains cost equivalent to in-person follow-up even when 100 percent of the mobile app follow-up care patients attend 1 in-person visit during the first month.

### Two-Way Sensitivity Analysis: Societal Perspective Costs With Varying Foregone Patient Leisure Time and Mobile Effectiveness

The patient’s wage was set between $11.28 (homemaker) and $26.71 (age/sex adjusted) per hour wage. The mobile effect was varied between a 90-96% success rate. Table 2 demonstrates how an incremental net benefit only favors (ie, produces a negative value) in-person follow-up if a 6 percentage point difference in effect exists between the two follow-up groups and the patient makes <$19 CAD per hour. This calculation uses a willingness-to-pay (WTP) of $100,000 USD ($109,970 CAD based on the current exchange rate) per quality adjusted life year (QALY), and a 0.04 QALY difference between no complication and minor skin infection. This is a high estimate previously reported in the literature [19].

**Table 2:** Two-way sensitivity analysis with varying patient lost leisure time and effectiveness of mobile app follow-up care.^a^

		Patient lost leisure time (CAD $)		
Mobile Effect		$33.84	$56.98	$80.12
INB^b^ @	Effect 0.96	198.75	236.70	274.65
INB @	Effect 0.94	110.77	148.72	186.67
INB @	Effect 0.92	22.80	60.75	98.70
INB @	Effect 0.90	−65.18	−27.23	10.72

^a^Willingness-to-pay (WTP)=$4398.80 CAD per effect based on $109,970 CAD per quality adjusted life year (QALY) and 0.04 QALY assigned to one superficial skin infection [19].

^b^INB: incremental net benefit

### Probabilistic Sensitivity Analysis

**Figure 2:**
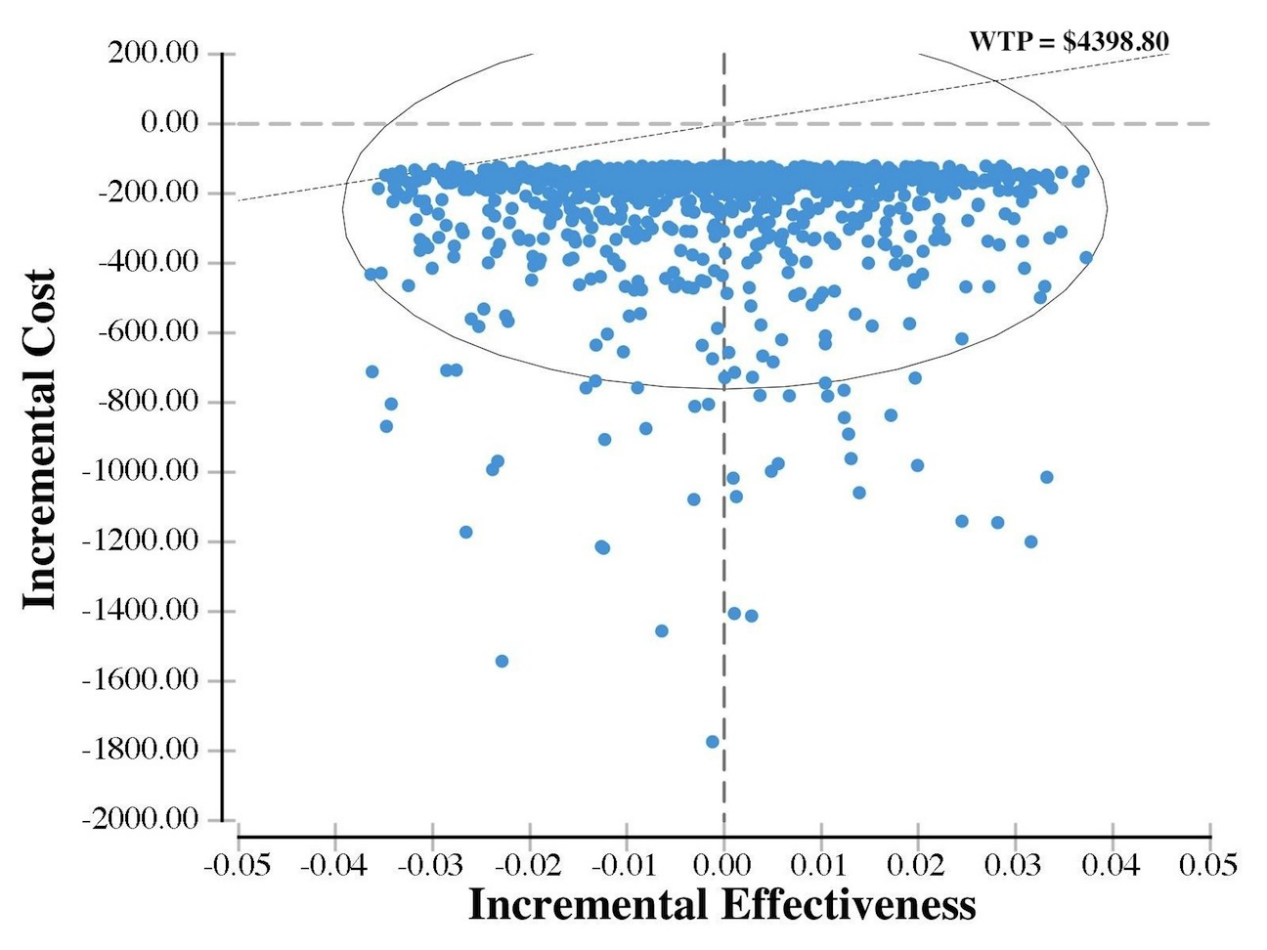
Graphical representation of probabilistic sensitivity analysis demonstrating the Incremental Cost-Effectiveness Ratio (ICER) values for mobile app versus in-person follow-up care.

To further explore the robustness of the base case results, a probabilistic sensitivity analysis was performed, based on random re-sampling (Monte Carlo simulation). In the analysis, a uniform distribution was allocated to the clinic cost (by +/− 20%), mobile and in-person follow-up effect (+/− 2 percentage points). A gamma distribution was applied to the patient wage. In 10,000 simulations, the mean societal cost of mobile app follow-up care was $135.78 CAD and in-person follow-up care was $383.55 CAD per patient. The large in-person follow-up care standard deviation of $211.80 CAD accurately reflects the variation in wage and travel time among patients. In all scenarios, mobile app follow-up care was cheaper than in-person follow-up care from a societal perspective. In approximately 50% of scenarios, the effectiveness of mobile app follow-up was less than in-person follow-up (see Figure 2). This was imposed by the distributions assigned. It is important to note, scenarios that are less effective and less costly can still be considered cost-effective. Again, using a WTP of $4398.80 CAD/superficial skin infection, mobile app follow-up care is the preferred strategy in 99.1% of scenarios.

## Discussion

### Principal Findings

Results from modeling cost-effectiveness show that mobile app follow-up care is cost-effective when compared to in-person follow-up care from a societal and health care system perspective. A detailed examination demonstrated that in the first month of follow-up care, an average of $136 CAD was spent in the mobile app follow-up care stream whereas $381 CAD was spent in the in-person follow-up care stream. The higher cost of in-person follow-up care is spread between the health care system and patient; however, the patient reaps the majority of the cost-savings from participating in mobile app follow-up care. This is demonstrated by comparing the societal and health care system perspective savings ($245 vs $38, respectively), as the patient savings are only captured in the societal perspective. This is an important finding as lower socioeconomic status is a known barrier to breast reconstruction [5]. The two-way sensitivity analysis demonstrates how these savings are maintained even when the patient makes a homemaker wage. Decreasing costs incurred to the patient, at least in the postoperative period, may improve access.

There is a deficit in Canadian policy promoting mobile phone communication between patients and providers. Ontarians cannot even renew a prescription over the phone, unless they choose to pay out-of-pocket for a normally insured service, because there is no telemedicine prescription renewal code [20]. This cost-effectiveness study is an important first step in demonstrating to health care administrators and policy decision-makers the benefits of investing in mobile app follow-up care. Mobile app follow-up care generates an incremental net benefit of $38 per patient from the perspective of the health care system. Decreasing the total number of in-person follow-up visits required has the potential to generate efficiency in one of two ways. Hospitals could choose to investment in smaller clinic spaces, decreasing the fixed and variable costs that accompany these spaces. Alternatively, hospitals could serve more patients in a given clinic space, including more new consultations. This is an important finding given the concern with long specialty wait times across Canada [21]. Orthopedic surgery and plastic surgery have the longest wait times. These two specialties perform a significant number of ambulatory surgeries and their patients in particular could benefit from mobile app follow-up care. Moreover, the number of patients that would benefit from mobile app follow-up care is growing yearly. At Women’s College Hospital, over 5000 elective ambulatory surgeries are performed each year. These numbers are small when you look at other neighboring hospitals, where over 20,000 ambulatory surgeries are performed annually. These numbers will continue to grow as we follow trends in the United States where currently 60 to 70% of the surgical procedures are performed in the ambulatory setting [22].

Mobile app follow-up transmits the same information as telephone follow-up care, but its obvious advantages include its asynchronous nature and autonomy from health care labor force to call, collect, and relay the patient data. The ease of use allows data to be collected multiple times during the 30-day follow-up period. In our pilot study, patients submitted questionnaire and surgical site photos every day for the first 2 weeks and once a week for the following 2 weeks. This provides richer data than could ever be achieved by telephone or in-person follow-up care. At this point in time, mobile app follow-up makes sense. Usage is ubiquitous throughout North America. Mobile phone penetration is approaching 90% in the United States, and smartphones are now considered the dominant mobile device [23]. As technology is an economy of scale, the potential for cost-savings increases with user uptake.

### Limitations

There are a few limitations to this study. Equivalency in the effectiveness of mobile app and in-person follow-up care is assumed based on observational studies of telephone questionnaire-based follow-up care from similar ambulatory surgery patient populations. There are no randomized control trials demonstrating equal effectiveness between mobile app and in-person follow-up care. From a clinical perspective, effect equivalence is intuitive because outcomes are dependent on patient and surgical factors (face validity).

This study did not compare the cost-effectiveness of telephone follow-up care to mobile app and in-person follow-up care. This is because most HTA agencies recommend comparing technology to usual care [10]. Mobile app and telephone follow-up care utilize the same standardized questionnaire tool; however, telephone follow-up care has obvious disadvantages including (1) the reliance on synchronous communication between the patient and health care professional, (2) no capacity to submit surgical site photography, and (3) a heavy dependence on human resources leading to higher costs.

### Conclusions

Mobile app follow-up care is suitably targeted to low-risk postoperative ambulatory patients. It can be cost-effective from a societal and health care system perspective. Mobile phone penetration is approaching 90% in the United States, and smartphones are now considered the dominant mobile device. Using a ubiquitous technological platform to reduce health care costs for patients and providers in an already large and growing patient population makes sense.

## Abbreviations

BYOD: bring-your-own-device
HTA: health technology assessment
ICER: incremental cost-effectiveness ratio
INB: incremental net benefit
QALY: quality adjusted life year
WCH: Women’s College Hospital
WTP: willingness-to-pay
